# Two-Step Concentration of Complex Water Samples for the Detection of Viruses

**DOI:** 10.3390/mps1030035

**Published:** 2018-09-10

**Authors:** Kata Farkas, James E. McDonald, Shelagh K. Malham, Davey L. Jones

**Affiliations:** 1 School of Natural Sciences, Bangor University, Bangor LL57 2UW, UK; j.mcdonald@bangor.ac.uk (J.E.M.); d.jones@bangor.ac.uk (D.L.J.); 2 School of Ocean Sciences, Bangor University, Menai Bridge LL59 5AB, UK; s.mahlam@bangor.ac.uk; 3 UWA School of Agriculture and Environment, University of Western Australia, Crawley 6009, Australia; Davey.jones@uwa.edu.au

**Keywords:** tangential flow ultrafiltration, virus precipitation, qPCR, enteric viruses, norovirus, mengovirus

## Abstract

The accurate detection and quantification of pathogenic viruses in water is essential to understand and reduce the risk of human infection. This paper describes a two-step method suitable for concentrating viruses in water and wastewater samples. The method involves a tangential flow ultrafiltration step that reduces the sample volume of 1–10 L to approximately 50 mL, followed by secondary precipitation using polyethylene glycol 6000, which reduces the volume to 1–4 mL. For method validation, water samples were spiked with different concentrations of enteric viruses, and viral recovery in the concentrates exceeded 10% in all experiments. The method is suitable for water samples with high and low salinity and turbidity, allowing an accurate comparison of viral titers in a diverse range of water types. Furthermore, the method has the potential to concentrate other pathogens, e.g., bacteria or protozoa. Hence, the use of this method can improve the holistic assessment of risks associated with wastewater-contaminated environments.

## 1. Introduction

Enteric viruses (causing gastroenteritis) and other viral pathogens can be found in wastewater and in wastewater-contaminated surface and groundwater reservoirs. As the infective doses of these agents are low, concentration is needed to accurately quantify viruses in environmental waters and determine public health risks. A great variety of methods are available for water concentration for the recovery of viruses in wastewater and environmental water; however, many of these are not suitable and/or have not been validated for high volumes of water samples or different water types. The most frequently used method for primary concentration of water samples is filtration using electronegative (EN) or electropositive (EP) filters [1,2]. During EN or EP filtration, the water sample passes through the filter, while the virus particles bind to the surface of the filter due to electrostatic forces. This method has been shown to be suitable for concentrating viruses in water; however, its use may be limited to low-turbidity samples due to filter clogging during filtration. Furthermore, the use of electronegative filters requires sample preconditioning (i.e., lowering the sample pH), whereas electropositive filters may not be suitable for high-salinity samples, and the elution of virus particles from the filters may be difficult as well [1]. 

Tangential flow ultrafiltration (TFUF) has been used for the concentration of a wide range of water samples for the detection of various pathogens [1,2,3,4,5]. The main advantage of the TFUF approach is that during filtration, the water flow takes place parallel to the membrane, hence membrane clogging is less frequent compared to dead-end ultrafiltration and EN and EP filtration. In general, TFUF enables 40–200× concentration, hence secondary concentration (filtration or precipitation) is often used to further reduce the sample volume [6,7,8].

**Figure 1:**
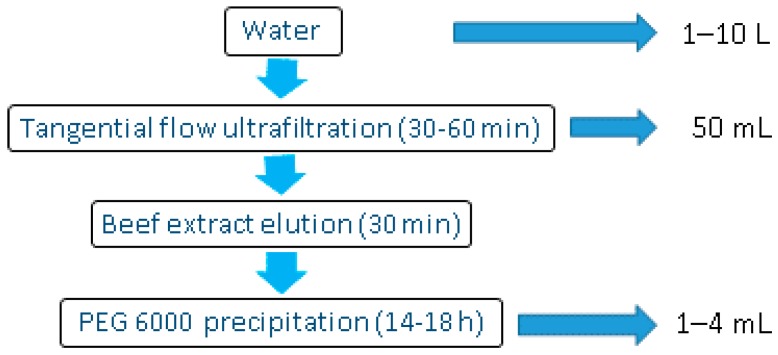
Stages of the tangential flow ultrafiltration–based two-step water concentration method for detection and quantification of enteric viruses in water and wastewater. PEG 600, polyethylene glycol 6000.

This method describes an efficient, accurate, and reproducible method for concentrating enteric viruses in surface water (fresh and seawater) and wastewater (treated and untreated) samples (Figure 1). The recommended starting volumes are 10 L for surface water and 1 L for wastewater samples. The first step of the method is a TFUF step using a 100 kDa cutoff modified polyethersulfone membrane. As described by others, the efficiency of the elution of viral particles from the membrane and the cleaning of the system is enhanced using sodium polyphosphate [4]. The final volume of the sample after TFUF is approximately 50 mL. In order to elute viral particles attached to solid matter in the primary concentrate, samples are further mixed with beef extract and then centrifuged. Then polyethylene glycol 6000 (PEG 6000) is added to the supernatant and viral particles are precipitated. These steps are based on a method for eluting and concentrating viral particles from sediment [9,10]. The resulting pellet contains the viral particles that can be eluted in phosphate-buffered saline (PBS), and the solution can be stored at −80 °C. The concentrate can be subjected to nucleic acid filtration, viral infectivity, or integrity assays.

## 2. Experimental Design

### 2.1. Materials

- Sodium polyphosphate (NaPP)/sodium hexametaphosphate (Sigma Aldrich, St. Louis, MO, USA, Cat. no. 305553)
- Lab-Lemco beef extract (Oxoid, Altrincham, Cheshire, UK, Cat. no. LP0029)
- Sodium nitrate (Sigma Aldrich, Cat. no. S8170)
- Polyethylene glycol 6000 (PEG 6000) (Sigma Aldrich, Cat. no. 81255)
- Sodium chloride (Sigma Aldrich, Cat. no. S7653)
- Phosphate-buffered saline (PBS), pH 7.4 (Gibco PBS tablets, Life Technologies, Carlsbad, CA, USA, Cat. no. 18912-014)
- Virkon^®^ solution (Lanxess, Cologne, Germany)
- 20% ethanol (Fisher Chemical #E/0650/17DF, Thermo Fisher Scientific, Waltham, MA, USA)
- 0.5 M HCl and 1 M NaOH for pH adjustment
- Optional: 30 µL mengovirus strain VMC0 solution (prepared according to ISO/TS150216-1:2013) with approximately 10^6^ mengovirus particles

### 2.2. Equipment

- KrosFlo^®^ Research IIi Tangential Flow Filtration System (Spectrum Labs, Phoenix, AZ, USA, Cat. no. SYR-U20-01N) or equivalent
- 100 kDa mPES MiniKros^®^ hollow fiber filter module (Spectrum Labs, USA, Cat. no. S02-E100-05-N)
- Silicone tubing #17 (Spectrum Labs, USA, Cat. no. ACTU-E17-25N) or equivalent
- Centrifuge (2500× *g* and 10,000× *g* at 4 °C)
- Pocket-sized pH meter (Ichiro Corporation, Kotoku, Tokyo, Japan, Cat. no. S2K992) or equivalent

## 3. Procedure

### 3.1. Tangential Flow Ultrafiltration (Time of Completion: 2–4 h)

#### 3.1.1. System Wash

**Figure 2:**
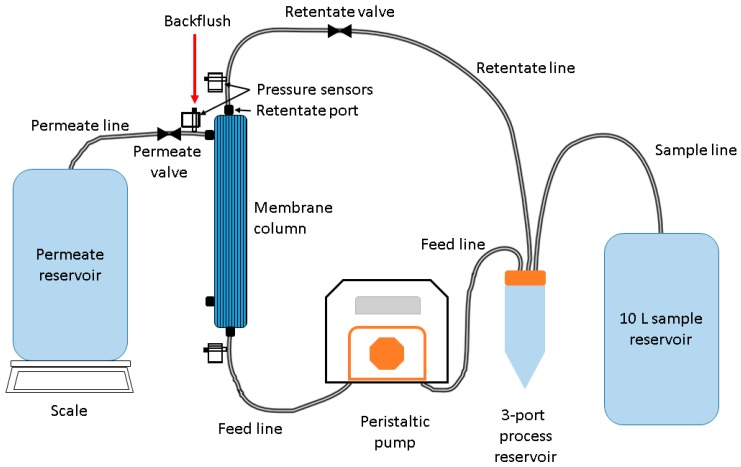
Schematic of tangential flow ultrafiltration setup.

1. Wash system (Figure 2) with 1 L 0.01% NaPP solution (0.1 g NaPP in 1 L deionized water) for 5 min (permeate closed) then leave the membrane in the solution for 30–60 min. Wash the membrane with the NaPP solution (permeate open) until the solution has been removed.

#### 3.1.2. Sample Filtration

**OPTIONAL STEP** Add approximately 10 µL mengovirus solution to the sample and mix. Save the rest of the mengovirus sample for control measurements.

2. Filter 10 L of surface water or 1 L wastewater at 1–1.6 L/min flow at a pressure of 5 psi (0.3 bar, 30 kPa) to achieve a permeate flow of 200–300 mL/min. Continue filtration until approximately 5 mL sample remains in the reservoir.

#### 3.1.3. Backwash, Recovery

3. Set the flow to 680 mL/min with no pressure applied and circulate the concentrate for 5 min with the permeate clamp closed.
4. Stop the pump, close penetrate and retentate valves.
5. Inject 20 mL 0.01% NaPP solution to penetrate pressure valve. Open retentate and wash with reverse flow.
6. Collect the concentrate from the system by introducing air through the retentate port. The final volume of concentrate is approximately 50 mL.

#### 3.1.4. Membrane Wash and Storage

7. Wash membrane with 250 mL Virkon^®^ solution after each sample by circulating the solution in the system (permeate closed) at low flow (400–800 mL/min). In order to reuse the membrane, immediately wash it with 150 mL 0.01% NaPP solution using the setup for the Virkon wash. Repeat until solution in the process reservoir is clear. Leave the membrane in the solution for at least 10 min prior to reuse.
8. For long-term storage, wash the membrane with 50 mL 20% ethanol solution using the setup for the Virkon wash. Repeat until solution is clear. Disassemble the system and store membrane in 20% ethanol solution at 4 °C.

### 3.2. Secondary Concentration (Time of Completion: 2.5 h + Overnight Incubation)

#### 3.2.1. Virus Elution

9. Add beef extract and NaNO_3_ to 50 mL concentrated water sample to reach final concentration of 3% *w*/*v* and 2 M, respectively. Adjust the pH to 5.5 using 0.5 M HCl.
10. Incubate at 50–90 rpm on ice for 30 min.
11. Centrifuge at 2500× *g* for 10 min, then transfer the supernatant to a new tube. Discard pellet. Adjust the pH of the solution to 7.5 using 1 M NaOH.

#### 3.2.2. Virus Precipitation

12. Add PEG 6000 and NaCl to reach final concentrations of 15% and 2% *w*/*v*, respectively. Mix to dissolve PEG 6000 and incubate at 4 °C for 14–18 h.
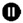
**PAUSE STEP** The solution may be stored at 4 °C for up to 2 days.
13. Centrifuge at 10,000× g for 30 min at 4 °C. Discard supernatant.
14. Dissolve pellet in 1–4 mL PBS (pH 7.4). The concentrate can be subjected to infectivity/integrity assays or nucleic acid extraction followed by real-time PCR quantification. Alternatively, viral nucleic acids can be extracted directly from the pellet.
  **OPTIONAL STEP** To estimate method recovery percentile, RNA from 10 µL mengovirus solution should be extracted and quantified.

## 4. Expected Results

For pilot validation, 2 L of deionized water was spiked with norovirus GII to reach final concentrations of 10^6^, 10^5^, 10^4^, 10^3^, 10^2^, and 10^1^ genome copies (gc)/L in duplicate. Samples were concentrated using the two-step concentration method. Viral RNA was extracted from the pellet (final volume: 50 µL) and quantified using qRT-PCR (sample volume: 2 µL) as described in Farkas et al. [10]. High recovery was observed in all norovirus concentrations (Table 1). The high deviations between replicates were a result of the limitations of the qPCR method used for quantification. The limit of quantification (LOQ) was 200 gc/L and the limit of detection (LOD) was approximately 50 gc/L. LOD and LOQ can be further lowered by reducing the RNA eluent volume and increasing the sample volume in the qRT-PCR reaction. 

For further validation, 10 L of surface water samples (river, estuarine, and sea) in triplicate were spiked with known concentrations of human enteric viruses (norovirus GII, sapovirus GI, hepatitis A virus, and human adenovirus type 40) and a mengovirus, which is often used as a process control for the extraction of enteric viruses from environmental matrices. The details of validation are described elsewhere [11]. Results showed that 10–100% recovery could be achieved in all sample types. In the same study, the usefulness of the method for wastewater samples was also investigated. Influent and effluent samples were taken in duplicate at four wastewater treatment plants. As the samples were expected to contain the target viruses, they were processed without spiking. High viral concentrations were observed, and the method showed great reproducibility. In subsequent samples spiked with mengovirus, at least 10% recovery was observed. No inhibition or cross-contamination between samples was observed. The method has also been successfully used for viral recovery from high volumes (50 L) of surface water for metagenomics applications [12]. 

### Applications and Recommendations

The method described above is suitable for concentrating many different water samples and hence suitable for conducting viral surveillance, tracking contamination sources, and describing viral ecology. The TFUF method described here is suitable for viral recovery from the mPES MiniKros^®^ hollow fiber filter (Spectrum Labs, Phoenix, AZ, USA); however, alternative recovery buffers may be used with different membranes. Furthermore, as the membrane used for the TFUF has a 100 kDa pore size, the method can potentially co-concentrate other microbes and protozoa as well, and hence the TFUF step of the concentration method enables accurate description of the microbial quality of a sample. In the current study, viral nucleic acids were directly extracted from the concentrated water samples; in addition, the concentrates are suitable for viral infectivity and capsid integrity assays as well [11]. For direct nucleic acid extraction, resuspension of the PEG precipitate is not necessary; nucleic acids can be extracted from the pellet. When PCR-based approaches are used for the quantification of viruses in the concentrate, the use of robust extraction and amplification methods is recommended, as organic matter that may interfere with the enzymes used for amplification are co-concentrated with viral particles. The addition of process control (e.g., mengovirus) to each sample to determine method efficiency is highly recommended.

## Figures and Tables

**Table 1:** Norovirus recovery and standard deviation (SD) in deionized water using the two-step concentration method.

Norovirus Concentration (gc/L)	Recovery Percentile (SD)
5.28 × 10^6^	78 (43)
4.72 × 10^5^	121 (2)
4.31 × 10^4^	99 (11)
3.03 × 10^3^	91 (45)
2.0 × 10^2^	100 (0)
